# Progressive FastICA Peel-Off and Convolution Kernel Compensation Demonstrate High Agreement for High Density Surface EMG Decomposition

**DOI:** 10.1155/2016/3489540

**Published:** 2016-08-25

**Authors:** Maoqi Chen, Ales Holobar, Xu Zhang, Ping Zhou

**Affiliations:** ^1^Biomedical Engineering Program, University of Science and Technology of China, Hefei, China; ^2^Guangdong Work Injury Rehabilitation Center, Guangzhou, China; ^3^Faculty of Electrical Engineering and Computer Science, University of Maribor, Maribor, Slovenia; ^4^Department of Physical Medicine and Rehabilitation, University of Texas Health Science Center at Houston, Houston, TX, USA; ^5^TIRR Memorial Hermann Research Center, Houston, TX, USA

## Abstract

Decomposition of electromyograms (EMG) is a key approach to investigating motor unit plasticity. Various signal processing techniques have been developed for high density surface EMG decomposition, among which the convolution kernel compensation (CKC) has achieved high decomposition yield with extensive validation. Very recently, a progressive FastICA peel-off (PFP) framework has also been developed for high density surface EMG decomposition. In this study, the CKC and PFP methods were independently applied to decompose the same sets of high density surface EMG signals. Across 91 trials of 64-channel surface EMG signals recorded from the first dorsal interosseous (FDI) muscle of 9 neurologically intact subjects, there were a total of 1477 motor units identified from the two methods, including 969 common motor units. On average, 10.6 ± 4.3 common motor units were identified from each trial, which showed a very high matching rate of 97.85 ± 1.85% in their discharge instants. The high degree of agreement of common motor units from the CKC and the PFP processing provides supportive evidence of the decomposition accuracy for both methods. The different motor units obtained from each method also suggest that combination of the two methods may have the potential to further increase the decomposition yield.

## 1. Introduction

The motor unit (which contains a spinal motor neuron, its axon, and the muscle fibers it innervates) is the final common pathway for neuromuscular control and provides a basic structure-function framework for neuromuscular system examination. Motor unit plasticity refers to motor unit adaptation or the ability of motor unit physical and functional changes as a result of activity, neurologic injury, age, rehabilitation training, and other factors. Motor unit plasticity can be assessed in different ways including by analyzing electromyogram (EMG) and muscle force output. Among various EMG signal processing methods, EMG decomposition provides a unique approach to observing the behavior of spinal motor neurons and its adaptation or alteration in human subjects by monitoring motor unit recruitment and firing rates.

EMG decomposition has been routinely performed with invasive needle electrodes [1–5]. The primary challenges of surface EMG decomposition arise from large number of active motor units, similar motor unit action potential (MUAP) waveforms for different motor units, and heavy MUAP superposition. With amplification technology developments, high density surface EMG relying on electrode arrays (comprised of up to hundreds of closely spaced tiny probes) has greatly advanced surface EMG decomposition. Various signal processing techniques using high density surface electrode arrays have been proposed for the decomposition purpose [6–9], among which the convolution kernel compensation (CKC) has achieved distinguished yield for high density surface EMG decomposition [10–12]. The CKC approach has been tested using both simulated and experimental surface EMG signals [13–15], including the “two-source” validation with simultaneous intramuscular EMG recordings [13].

Very recently, we have developed a progressive FastICA peel-off (PFP) framework for high density surface EMG decomposition and tested this novel framework with both simulated and experimental surface EMG signals [16]. Given that both the CKC and PFP methods are designed for high density surface EMG recordings, they can be applied to the same set of data. This provides a strategy to assess the decomposition performance for both methods by comparing the discharge instants of the common motor units from independent CKC and PFP decompositions. The objective of the current study was to use such a strategy to compare the decomposition yield from the two different methods. We hypothesized that when processing the same set of high density surface EMG signals, high agreement can be achieved when comparing the decomposition results, thus providing supportive evidence of the decomposition performance for both CKC and PFP methods.

## 2. Methods

### 2.1. Data Model

Different from most of the other blind source separation technologies in surface EMG, both PFP and CKC use a shift-invariant model to describe multichannel surface EMG signal [10, 16], which allows MUAP shapes of a specific motor unit that vary in different channels but share the same discharge instants. Assuming *N* active motor units recorded by *M* surface electrodes: **x** = [*x*_1_, *x*_2_,…,*x*_*M*_]^*T*^, the signal on each channel can be described as

(1) xit=∑j=1N∑ τ=0L−1aijτsjt−τ+nit;i=1,2,…,M,  t=0,1,…,T.

In CKC, (1) can also be viewed as a convolutive linear time-invariant multiple-input multiple-output (MIMO) model, where *n*_*i*_(*t*) represents the additive white, zero-mean Gaussian noise in the* i*th channel. Each model input *s*_*j*_(*t*) = ∑_*k*_ *δ*(*t* − *T*_*j*_(*k*)) is a sparse binary motor unit discharge pattern (i.e., its values are either 0 or 1) that indicates whether the* j*th motor unit discharges at a specific time *t*. *T*_*j*_(*k*) is the *k*th discharge time of the *j*th motor unit, whereas *δ* represents Dirac Delta function. The channel response *a*_*ij*_ stands for the waveform of the *j*th motor unit in the *i*th channel; *L* is the length of the waveform. It is assumed that *T*_*j*_(*k* + 1) − *T*_*j*_(*k*) > *L* for each *k*.

The model in (1) can be rewritten in matrix form:

(2) $$\begin{array}{c} x\left(\;t\;\right)=A\overset{-}{s}\left(\;t\;\right)+n\left(\;t\;\right), \end{array}$$

where $\overset{-}{s}\left(t\right)={\left[s_{1}\left(t\right),s_{1}\left(t-1\right),\ldots ,s_{1}\left(t-L+1\right),\ldots ,s_{N}\left(t\right),\ldots ,s_{N}\left(t-L+1\right)\right]}^{T}$ stands for an extended form of a sample vector **s**(*t*) and **n**(*t*) = [*n*_1_(*t*), *n*_2_(*t*),…,*n*_*M*_(*t*)]^*T*^ is a noise vector. The unknown matrix **A** comprises all the MUAPs as detected by the different surface electrodes (for details, please refer to [10, 16]).

### 2.2. Introduction of CKC and PFP

The CKC method first blindly estimates the cross-correlation vector between the discharge pattern of one motor unit and the EMG measurements. Then the unknown mixing matrix** A **(i.e., the convolution kernel) is partially compensated by calculating an estimation of the discharge patterns of this motor unit using the estimated cross-correlation vector and the correlation matrix of the EMG signal. As the convolution kernel is compensated gradually a number of motor units can be estimated. More details on CKC processing can be found in [10].

The PFP framework can be viewed as a process of progressively expanding the set of spike trains. In the framework, FastICA is used to estimate motor unit spike trains. A “peel-off” procedure is employed to estimate the MUAPs of all the identified motor units and subtract them from the original signal. Such a procedure mitigates the effect of the already identified motor units on the FastICA convergence, so more motor units can emerge when processing the residual signal. In order to ensure the reliability of the decomposition, a constrained FastICA is applied to assess the newly extracted discharge patterns and correct possible erroneous or missed spikes. These features work together to promote the decomposition yield. More details on PFP processing can be found in [16].

### 2.3. Data Description

The surface EMG signals used for testing the proposed framework were acquired from the first dorsal interosseous (FDI) muscle of nine healthy subjects. The procedures were approved by the local Institutional Review Board. All the subjects gave their written consent before the experiment. Subjects were seated upright in a mobile Biodex chair (Biodex, Shirley, NY). A standard 6 degrees of freedom load cell (ATI Inc., Apex, NC) setup was used to accurately record the isometric contraction force of the FDI muscle during index finger abduction. Standard procedures were followed to minimize spurious force contributions from unrecorded muscles as described in [17]. Surface EMG signals were recorded using a flexible two-dimensional 64-channel (8 × 8, individual recording probe 1.2 mm in diameter, center-to-center distance of 4 mm) surface electrode array (TMS International BV, Netherlands). The maximum voluntary contraction (MVC) was first measured; after that, each subject was asked to generate an isometric contraction force of the FDI muscle at different contraction levels. The subject was asked to maintain the force as stable as possible for at least 3 s (preferably more than 5 s). The actual percent MVC for each contraction was calculated afterwards by normalizing the force measurement (averaged from the stable force period) to each subject's MVC. A Refa128 amplifier (TMS International BV, Netherlands) was used to record surface EMG signals. The signals were sampled at 2 kHz per channel, with a bandpass filter setting at 10–500 Hz. Totally 91 experimental surface EMG signals (35 ± 27% MVC, range: ~1% to ~100% MVC) were decomposed by PFP and CKC, respectively. The two decomposition processes were independent of each other and they were operated by two different operators. The decomposition by CKC was first processed by an automatic program, and a manual motor unit selecting process was used to ensure the reliability of the results. In particular, recently introduced pulse-to-noise ratio (PNR) metrics [18] has been employed to assess the accuracy of motor unit identification and only the motor units with PNR ≥ 30 dB (sensitivity in identification of motor unit discharges ≥ 90%) were kept whereas all the other motor units were discarded. For the PFP, manual monitoring was used to guarantee the reliability when using constrained FastICA to assess the identified spike trains.

### 2.4. Data Analysis

The matching rate (MR) was calculated to precisely measure the matching degree of the commonly identified motor units from the two decomposition methods. For each common motor unit, the matching rate between two decompositions was calculated as

(3) $$\begin{array}{c} \text{MR}=\frac{2\cdot N_{\text{COM}}}{N_{\text{CKC}}+N_{\text{PFP}}}\cdot 100\%, \end{array}$$

where *N*_COM_ stands for the number of discharges of a motor unit that were identified by both decomposition techniques (i.e., the number of corresponding discharges within time tolerance of ±1 ms). *N*_CKC_ and *N*_PFP_ are the total number of discharges which were identified by CKC and PFP, respectively. Note that if either of the two spike trains is considered as the “standard” spike train, MR is actually an *F*1-score measure [19]. In this study we consider a motor unit as a common one only when MR between the two decomposition methods is higher than 90%.

A cross-correlation function method introduced in [16] was used to facilitate the identification of coupling discharge spike trains from the two decomposition algorithms and calculate MR. The following parameters were calculated:

(4) ρi∗=maxj⁡maxt⁡RsC,i,sP,jtRsC,i,sC,i0·RsP,j,sP,j0,ji∗=arg⁡maxj⁡maxt⁡RsC,i,sP,jtRsC,i,sC,i0·RsP,j,sP,j0,ti∗=arg⁡maxt⁡RsC,i,sP,ji∗tRsC,i,sC,i0·RsP,ji∗,sP,ji∗0,

where *R*_·,·_(*t*) represents cross-correlation function, *s*_*C*,*i*_ stands for the *i*th spike train identified from CKC, and *s*_*P*,*j*_ is the *j*th spike train identified from PFP. *ρ*_*i*_^*∗*^ is the maximum cross-correlation coefficient between *s*_*C*,*i*_ and *s*_*P*,*j*_. If *ρ*_*i*_^*∗*^ ≥ 0.3, we accepted potential existence of a spike train coupling between *s*_*C*,*i*_ and *s*_*P*,*j*_. For the identified “coupling”, *j*_*i*_^*∗*^ was used as the indicator of the corresponding spike train and the value of MR was used to determine whether the two spike trains really correspond or not. For this purpose, the corresponding delay *t*_*i*_^*∗*^ has been estimated and *s*_*C*,*i*_ and *s*_*P*,*j*_ aligned in time. After such a time shift, MR has been calculated as defined in (3).

## 3. Results

**Figure 1:**
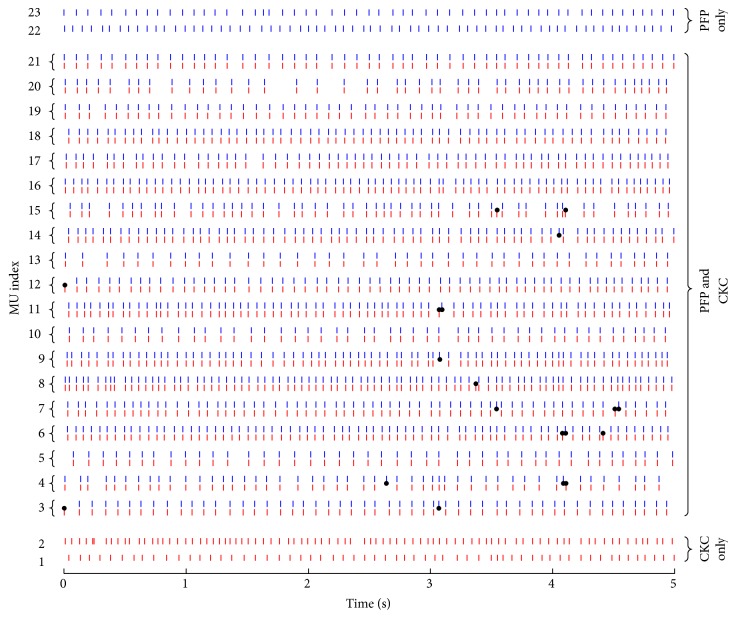
An example of discharge instants for motor units identified from an isometric contraction at ~18% MVC. The red spike trains represent the results obtained by the CKC, and the blue ones represent the results obtained by the PFP. Black dots represent the locations where the two methods generated inconsistent discharge instants.

Figure 1 shows an example of discharge instants for motor units identified from an isometric contraction at the level of approximately 18% MVC. The red spike trains represent the results obtained by the CKC, and the blue ones are the results obtained by the PFP. In this example, 19 common motor units were identified, whose discharge patterns are aligned together in the figure. Black dots represent few locations where the two methods generated inconsistent discharge instants. In addition, each method also identified two different motor units, respectively, as shown in the figure.

Ninety-one trials of 9 subjects were processed with signal duration ranged from 3.2 to 11.2 s (7.9 ± 1.8 s), from which the matching rate was calculated. There were a total of 1477 motor units identified from the two methods, including 969 common motor units. On average, 10.6 ± 4.3 common motor units were identified from each trial, which showed a very high matching rate of 97.85 ± 1.85%. We did not observe a clear dependence of the number of common motor units and the matching rate on the contraction level. In addition to the common motor units which accounted for the majority of the decomposition yield, the two methods also identified a relatively small number of different motor units, such as those demonstrated in Figure 1, where 4 different motor units were identified from the two methods. Across the 91 trials, there were 5.6 ± 2.8 different motor units identified per trial from the two methods.

## 4. Discussion

Both CKC and PFP methods are designed for high density surface EMG decomposition, using blind source separation approaches based on a sparse shift-invariant model. The sparsity assumption for the motor unit discharge patterns ensures the algorithm can obtain sufficient information to separate the motor units. Unlike most of other decomposition methods primarily relying on MUAP template matching, the two algorithms focus on the underlying discharge patterns (i.e., the sparse components) in the EMG signal. The key iterative rules of the two algorithms also have a similar structure. Because of these similarities, the two methods achieved high agreement for high density surface EMG decomposition, as demonstrated in this study.

When comparing the decomposition yield from CKC and PFP methods, we only focused on the motor unit discharge instants, from which the MUAP waveforms can be estimated using spike triggered averaging (actually, during the PFP decomposition, the MUAP waveforms already emerge). Thus, if high agreement can be achieved in motor unit discharge instants between the two methods, high agreement in MUAP waveforms can also be expected.

In addition to the majority of common motor units, the two methods also identified a relatively small portion of different motor units. This might be due to differences between the PFP and CKC methods, such as in cost function and motor unit searching strategy (in dealing with the local convergence problem in gradient-based algorithm). For example, the CKC acts on original signal and each time the initial value is properly selected (at motor unit discharge instants) to ensure that the algorithm can converge to reliable results. Furthermore, the CKC adopts a probabilistic strategy, by blindly running the algorithm multiple times (e.g., 100 runs) and Gram-Schmidt orthogonalization of separation vectors to allow the algorithm to have sufficient probability to find those difficult convergent solutions and finally integrate all the results. Conversely, PFP adopts a different deflation strategy. When new solutions are obtained, the algorithm uses the information from discharge patterns of the already identified or validated motor units to estimate their MUAP trains and subtract them from the original signal and then applies FastICA to the residual signal to search other motor units. Such a deflation strategy mitigates the effect of the already identified motor units on the FastICA convergence, so extra motor units can emerge. However, it may lead to a cumulative error problem; that is, the early estimation error will be accumulated and magnified in the later process (this is why the constrained FastICA is used to ensure the accuracy of the identified spike trains).

Given that the CKC based surface EMG decomposition has been extensively validated in different situations [13–15], the high degree of agreement of common motor units between the decomposition results to some extent supports the accuracy of the PFP decomposition (and the accuracy of the CKC decomposition as well). To further confirm the accuracy of the PFP decomposition, simultaneous intramuscular EMG recording is necessary so a two-source validation can be performed.

Finally, it is noteworthy that some components of one method can be combined with the other. For example, the PFP can use the probability strategy (as used in the CKC) during each iteration to achieve more solutions. The CKC can adopt the MUAP estimation and the motor unit spike train validation mechanism similar to the constrained FastICA. Such a combination of CKC and PFP methods needs further investigation and might have a potential to increase the decomposition yield.

## References

[1] Stashuk D. (2001). EMG signal decomposition: how can it be accomplished and used?. *Journal of Electromyography and Kinesiology*.

[2] Parsaei H., Stashuk D. W., Rasheed S., Farkas C., Hamilton-Wright A. (2010). Intramuscular EMG signal decomposition. *Critical Reviews in Biomedical Engineering*.

[3] McGill K. C., Lateva Z. C., Marateb H. R. (2005). EMGLAB: an interactive EMG decomposition program. *Journal of Neuroscience Methods*.

[4] De Luca C. J. (1995). Decomposition of the EMG signal into constituent motor unit action potentials. *Muscle and Nerve*.

[5] Nawab S. H., Wotiz R. P., De Luca C. J. (2008). Decomposition of indwelling EMG signals. *Journal of Applied Physiology*.

[6] Kleine B. U., van Dijk J. P., Lapatki B. G., Zwarts M. J., Stegeman D. F. (2007). Using two-dimensional spatial information in decomposition of surface EMG signals. *Journal of Electromyography and Kinesiology*.

[7] Gligorijević I., van Dijk J. P., Mijović B., van Huffel S., Blok J. H., De Vos M. (2013). A new and fast approach towards sEMG decomposition. *Medical and Biological Engineering and Computing*.

[8] Garcia G. A., Okuno R., Azakawa K. (2005). A decomposition algorithm for surface electrode-array electromyogram. *IEEE Engineering in Medicine and Biology Magazine*.

[9] Theis F. J., García G. A. (2006). On the use of sparse signal decomposition in the analysis of multi-channel surface electromyograms. *Signal Processing*.

[10] Holobar A., Zazula D. (2007). Multichannel blind source separation using convolution kernel compensation. *IEEE Transactions on Signal Processing*.

[11] Holobar A., Zazula D. (2004). Correlation-based decomposition of surface electromyograms at low contraction forces. *Medical and Biological Engineering and Computing*.

[12] Holobar A., Farina D., Gazzoni M., Merletti R., Zazula D. (2009). Estimating motor unit discharge patterns from high-density surface electromyogram. *Clinical Neurophysiology*.

[13] Marateb H. R., McGill K. C., Holobar A., Lateva Z. C., Mansourian M., Merletti R. (2011). Accuracy assessment of CKC high-density surface EMG decomposition in biceps femoris muscle. *Journal of Neural Engineering*.

[14] Holobar A., Minetto M. A., Botter A., Negro F., Farina D. (2010). Experimental analysis of accuracy in the identification of motor unit spike trains from high-density surface EMG. *IEEE Transactions on Neural Systems and Rehabilitation Engineering*.

[15] Merletti R., Holobar A., Farina D. (2008). Analysis of motor units with high-density surface electromyography. *Journal of Electromyography and Kinesiology*.

[16] Chen M., Zhou P. (2016). A novel framework based on FastICA for high density surface EMG decomposition. *IEEE Transactions on Neural Systems and Rehabilitation Engineering*.

[17] Li X., Suresh A., Zhou P., Rymer W. Z. (2013). Alterations in the peak amplitude distribution of the surface electromyogram poststroke. *IEEE Transactions on Biomedical Engineering*.

[18] Holobar A., Minetto M. A., Farina D. (2014). Accurate identification of motor unit discharge patterns from high-density surface EMG and validation with a novel signal-based performance metric. *Journal of Neural Engineering*.

[19] Goutte C., Gaussier E., Losada D. E., Fernández-Luna J. M. (2005). A probabilistic interpretation of precision, recall and *F*-score, with implication for evaluation. *Advances in Information Retrieval*.

